# Safety of dostarlimab in combination with chemotherapy in patients with primary advanced or recurrent endometrial cancer in a phase III, randomized, placebo-controlled trial (ENGOT-EN6-NSGO/GOG-3031/RUBY)

**DOI:** 10.1177/17588359241277656

**Published:** 2024-09-28

**Authors:** Annika Auranen, Matthew A. Powell, Vladyslav Sukhin, Lisa M. Landrum, Graziana Ronzino, Joseph Buscema, Dirk Bauerschlag, Roy Lalisang, David Bender, Lucy Gilbert, Amy Armstrong, Tamar Safra, Nicole Nevadunsky, Alexandra Sebastianelli, Brian Slomovitz, Kari Ring, Robert Coleman, Iwona Podzielinski, Ashley Stuckey, Michael Teneriello, Sarah Gill, Bhavana Pothuri, Lyndsay Willmott, Sudarshan Sharma, Christine Dabrowski, Grace Antony, Shadi Stevens, Mansoor Raza Mirza, Evelyn Fleming

**Affiliations:** Tays Cancer Centre and FICAN Mid, Tampere University and Tampere University Hospital, Pirkanmaa Hospital District, FM3 2.krs. Biokatu 10, Tampere 33900, Finland; National Cancer Institute Sponsored NRG Oncology, Washington University School of Medicine, St Louis, MO, USA; Grigoriev Institute for Medical Radiology and Oncology National Academy of Medical Sciences of Ukraine, Kharkiv, Ukraine; Indiana University Health and Simon Cancer Center, Indianapolis, IN, USA; Department of Oncology, Ospedale “Vito Fazzi,” Lecce, Italy; Gynecologic Oncology, Arizona Oncology, Tucson, AZ, USA; University Hospital Schleswig-Holstein, Campus Kiel, Kiel, Germany; Department of Internal Medicine, Maastricht UMC Comprehensive Cancer Center, GROW-School of Oncology and Reproduction, Maastricht University Medical Center, Maastricht, The Netherlands; Department of Obstetrics and Gynecology, University of Iowa, Iowa City, IA, USA; Division of Gynecologic Oncology, Gerald Bronfman Department of Oncology, Research Institute—McGill University Health Centre, McGill University, Montreal, QC, Canada; Division of Gynecologic Oncology, University Hospitals Cleveland Medical Center, Case Comprehensive Cancer Center, Cleveland, OH, USA; Department of Oncology, Tel Aviv Sourasky Medical Center, and Faculty of Medicine, Tel Aviv University, Tel Aviv, Israel; Department of Obstetrics, Gynecology, and Women’s Health, Montefiore Medical Center, Bronx, NY, USA; CHU de Québec-Université Laval, Québec, QC, Canada; Department of Gynecologic Oncology, Mount Sinai Medical Center, and Department of Obstetrics and Gynecology, Florida International University, Miami Beach, FL, USA; University of Virginia Health System, Charlottesville, VA, USA; Texas Oncology, US Oncology Network, The Woodlands, TX, USA; Department of Gynecologic Oncology, Parkview Health, Fort Wayne, IN, USA; Women and Infants Hospital of Rhode Island, Providence, RI, USA; US Oncology Research, The Woodlands, TX, USA; St. Joseph’s/Candler Gynecologic Oncology and Surgical Specialists, Candler Hospital, Savannah, GA, USA; GOG Foundation and Departments of Obstetrics/Gynecology and Medicine, Division of Gynecologic Oncology, Laura and Isaac Perlmutter Cancer Center, NYU Langone Health, New York, NY, USA; Arizona Oncology, Phoenix, AZ, USA; Department of Obstetrics/Gynecology, AMITA Health Adventist Medical Center, Hinsdale, IL, USA; GSK, Collegeville, PA, USA; GSK, London, UK; GSK, London, UK; Rigshospitalet, Copenhagen University Hospital, Copenhagen, Denmark; Nordic Society of Gynaecologic Oncology Clinical Trial Unit, Copenhagen, Denmark; Division of Gynecologic Oncology, Norris Cotton Cancer Center, Dartmouth-Hitchcock Medical Center, Lebanon, NH, USA

**Keywords:** dostarlimab, dostarlimab plus chemotherapy, endometrial cancer, immune checkpoint inhibitor, safety

## Abstract

**Background:**

In Part 1 of the phase III RUBY trial (NCT03981796) in patients with primary advanced or recurrent endometrial cancer (EC), dostarlimab plus carboplatin–paclitaxel (CP) significantly improved progression-free survival and overall survival compared with CP alone. Limited safety data have been reported for the combination of immunotherapies plus chemotherapy in this setting.

**Objectives:**

The objective of this analysis was to identify the occurrence of treatment-related adverse events (TRAEs) and immune-related adverse events (irAEs) and to describe irAE management in Part 1 of the RUBY trial.

**Design:**

RUBY is a phase III, randomized, double-blind, multicenter study of dostarlimab plus CP compared with CP alone in patients with primary advanced or recurrent EC.

**Methods:**

Patients were randomized 1:1 to dostarlimab 500 mg, or placebo, plus CP every 3 weeks for 6 cycles, followed by dostarlimab 1000 mg, or placebo, every 6 weeks for up to 3 years. Adverse events (AEs) were assessed according to Common Terminology Criteria for Adverse Events, version 4.03.

**Results:**

The safety population included 487 patients who received ⩾1 dose of treatment (241 dostarlimab plus CP; 246 placebo plus CP). Treatment-emergent AEs were experienced by 100% of patients in both arms. TRAEs occurred in 97.9% of the dostarlimab arm and 98.8% of the placebo arm.

The most common TRAEs occurred at similar rates between arms and were mostly low grade. IrAEs occurred in 58.5% of patients in the dostarlimab arm and 37.0% of patients in the placebo arm. Dostarlimab- or placebo-related irAEs were reported in 40.7% of patients in the dostarlimab arm and 16.3% of the placebo arm.

**Conclusion:**

The safety profile of dostarlimab plus CP was generally consistent with that of the individual components. Dostarlimab plus CP has a favorable benefit–risk profile and is a new standard of care for patients with primary advanced or recurrent EC.

**Trial registration:**

NCT03981796.

## Plain language summary

Safety of dostarlimab plus carboplatin-paclitaxel compared with carboplatin-paclitaxel in primary advanced or recurrent endometrial cancer

For many years, patients with primary advanced or recurrent endometrial cancer were treated with chemotherapy, specifically with a combination of carboplatin and paclitaxel. Recently, new treatments called immune checkpoint inhibitors have been used to treat endometrial cancer. Dostarlimab, an immune checkpoint inhibitor, is being tested to treat many types of cancer, including endometrial cancer. In the RUBY trial, a combination of dostarlimab plus chemotherapy was compared with chemotherapy alone as treatment for primary advanced or recurrent endometrial cancer. Results showed that patients treated with dostarlimab plus chemotherapy had a lower risk of their cancer becoming worse and a lower risk of dying. Results in this article describe the safety of dostarlimab plus chemotherapy compared with chemotherapy alone. All patients in the RUBY trial experienced at least one adverse event (an undesired effect that happens while receiving treatment or shortly after stopping treatment); most were determined to be caused by the cancer treatments. No differences in the frequency of the overall cancer treatment-related adverse events were seen in patients who received dostarlimab plus chemotherapy compared with those patients who received chemotherapy alone. Some patients experienced an immune-related adverse event. These are a specific type of undesired effect that can occur when patients are treated with immune checkpoint inhibitors. Immune-related adverse events occurred more frequently in patients who received dostarlimab plus chemotherapy than in those who received chemotherapy alone. Physicians were generally able to treat the immune-related adverse events, and only a low percentage of patients discontinued treatment because they experienced an immune-related adverse event. The types of adverse events seen were similar to a combination of those seen in patients who received dostarlimab alone or patients who received chemotherapy alone as treatment for endometrial cancer. Dostarlimab plus chemotherapy is a new standard of care for patients with primary advanced or recurrent endometrial cancer.

## Introduction

Endometrial cancer (EC) is the sixth most common cancer among women worldwide.^1^ Globally, it is the second most common gynecologic cancer after cervical cancer, and in developed countries in which incidence rates are particularly high (i.e. North America, Europe, and Oceania), it is the most common gynecologic cancer.^1–4^ Moreover, the global age-standardized incidence rate of newly diagnosed EC increased by 0.69% since 1990; the aging population and increasing levels of obesity may potentially be contributors to this increased incidence.^2–7^

Despite improvements in mortality for many cancer types in recent years, the mortality rates for EC have been increasing worldwide.^7–9^ Carboplatin–paclitaxel (CP) has long been the standard-of-care first-line treatment for primary advanced or recurrent EC^10^; however, long-term outcomes have remained poor, with median overall survival (OS) durations of 3 years or less.^11,12^

Dostarlimab is a humanized monoclonal antibody that binds to programmed cell death receptor 1 (PD-1) and blocks the interaction of PD-1 with both of its ligands, PD-L1 and PD-L2.^13,14^ Dostarlimab plus CP followed by dostarlimab monotherapy is approved in multiple countries for adult patients with primary advanced or recurrent EC that is mismatch repair deficient (dMMR)/microsatellite instability-high (MSI-H).^15–17^ Recently, dostarlimab plus CP followed by dostarlimab monotherapy was approved in the United States for all adult patients with primary advanced or recurrent EC.^18^ These approvals were based on results from Part 1 of the phase III RUBY trial (NCT03981796), which met its primary endpoints for progression-free survival (PFS) in the dMMR/MSI-H and overall populations and OS in the overall population of patients with primary advanced or recurrent EC.^19,20^ Dostarlimab, in combination with CP, is the first immunotherapy to be approved in the first-line setting for the treatment of patients with dMMR/MSI-H primary advanced or recurrent EC.

Dostarlimab monotherapy reported a safety profile consistent with other anti-PD-(L)1 drugs.^21^ In the phase I GARNET trial, most treatment-related adverse events (TRAEs) were low grade, and few resulted in treatment discontinuation.^22,23^ To date, limited safety data have been reported for anti-PD-(L)1 drugs in combination with CP in primary advanced or recurrent EC.

Here, we present the safety data of dostarlimab in combination with CP from Part 1 of the RUBY trial (NCT03981796). The evaluations in this manuscript aim to identify the occurrence of TRAEs and immune-related adverse events (irAEs), to describe irAE management, and to advance understanding of the safety profile of dostarlimab in combination with CP versus dostarlimab monotherapy.

## Methods

### Participants

Patients eligible for participation in the RUBY trial were aged ⩾18 years and had primary advanced or recurrent EC that was histologically or cytologically confirmed and was not amenable to curative therapy. Full details of the inclusion and exclusion criteria have been published.^19^ In brief, the key inclusion criteria were Response Evaluation Criteria in Solid Tumors (RECIST) version 1.1-evaluable primary advanced EC of International Federation of Gynecology and Obstetrics stage IIIA, IIIB, or IIIC1 (as assessed by the investigator); primary advanced EC of stage IIIC1 (with carcinosarcoma, clear-cell, serous, or mixed histologic characteristics); or primary advanced stage IIIC2, or stage IV disease, regardless of the presence of disease that could be evaluated or measured; or disease that was either in its first recurrence without prior treatment with systemic therapy or had been treated with neoadjuvant or adjuvant systemic therapy and had recurred or progressed at least 6 months after completion of treatment (first recurrence). Availability of tumor samples for the assessment of MMR and microsatellite status was required.

### Study design and interventions

The RUBY trial is a phase III, randomized, double-blind, multicenter study. In Part 1 of the RUBY trial, patients with primary advanced or recurrent EC were randomly assigned in a 1:1 ratio to receive either dostarlimab or placebo. Assignment was stratified according to MMR/MSI status, previous external pelvic radiotherapy, and disease status.

Dostarlimab (500 mg) or placebo was administered intravenously every 3 weeks for 6 cycles with carboplatin (area under the curve 5 mg/mL/min) and paclitaxel (175 mg/m^2^) chemotherapy (referred to as the chemotherapy period), followed by 1000 mg of dostarlimab or placebo every 6 weeks as monotherapy (referred to as the monotherapy period).^19^ Treatment was for up to 3 years or until the occurrence of progressive disease, toxicity, withdrawal of consent, investigator’s decision, or death. Continued treatment with dostarlimab or placebo beyond 3 years could be considered after discussion between the study sponsor and the investigator.

The RUBY trial was registered at ClinicalTrials.gov (NCT03981796) and was conducted in accordance with the principles of the Declaration of Helsinki, Good Clinical Practice guidelines, and all applicable local laws. All patients provided written informed consent for participation. The reporting of this study conforms to the CONSORT guidelines (Supplemental File).^24^

### Assessments

The primary endpoints of the RUBY trial were investigator-assessed PFS (per RECIST version 1.1) in the dMMR/MSI-H and overall populations and OS in the overall population; both have been met.^19,20^ Safety outcomes were a prespecified secondary endpoint of the trial.

Safety was assessed through monitoring adverse events (AEs) according to the Common Terminology Criteria for Adverse Events (CTCAE) version 4.03. AE reporting, dose interruptions or delays, and concomitant medications were recorded using an electronic case reporting form.

Treatment-emergent AEs (TEAEs) were defined as any event that occurred on or after the start of treatment through 90 days after the last dose of study treatment (or until the start of alternate anticancer therapy, whichever occurred earlier) that was not present prior to the initiation of study treatment, or any event already present that worsened in either intensity or frequency after exposure to study treatment. TRAEs were defined by the investigator as events for which a causal relationship between any study treatment (dostarlimab, carboplatin, and/or paclitaxel) and the AE was a reasonable possibility. IrAEs were classified as CTCAE grade ⩾2 from a predefined list of preferred terms. IrAEs were also categorized as either treatment-emergent (TE irAE) or treatment-related (TR irAE); in addition, those TR irAEs specifically related to dostarlimab or placebo as reported by the investigator were also determined. Frequency, onset, and duration of TEAEs and irAEs were evaluated.

Management of irAEs was conducted per protocol specifications; the joint American Society of Clinical Oncology and National Comprehensive Cancer Network guidelines for the diagnosis and management of irAEs was used as a supplement for management.^21^

### Statistical analyses

TEAEs were summarized by treatment group and reported as number (percentage) of patients. Tabulation was also performed by severity and relationship to study treatment. A worst-case scenario was applied to missing treatment-relatedness data, with missing data categorized as related to treatment. The time to onset and duration of the first occurrence of TEAEs and irAEs were estimated and summarized using summary statistics (mean, standard deviation, median, and range). Duration was defined as the time from onset of any TEAE or irAE to the first time the patient was free of any such event. It required at least a 1-day gap between the resolution of all events from the first course to the onset of the second course, if applicable. All calculations were performed using SAS software version 9.4 (SAS Institute, Inc., Cary, NC, USA).

## Results

### Patients

In total, 494 patients were randomized 1:1 to receive either dostarlimab plus CP (dostarlimab arm) or placebo plus CP (placebo arm) in the RUBY trial, of whom 7 did not receive treatment. Thus, the safety population included 241 patients in the dostarlimab plus CP arm and 246 patients in the placebo plus CP arm who received at least 1 dose of treatment.

Full baseline demographic and clinical details have been previously published; no notable between-arm differences were noted.^19^ Patients in the dostarlimab and placebo arms were predominantly white (77.1% and 76.7%), had a respective median age of 64 and 65 years, a respective median body mass index of 30.8 and 32.8 kg/m^2^, and more than half (54.7% and 54.6%) had endometrioid EC. Overall, 21.6% in the dostarlimab arm and 26.1% in the placebo arm had dMMR/MSI-H EC.

**Figure 1.**
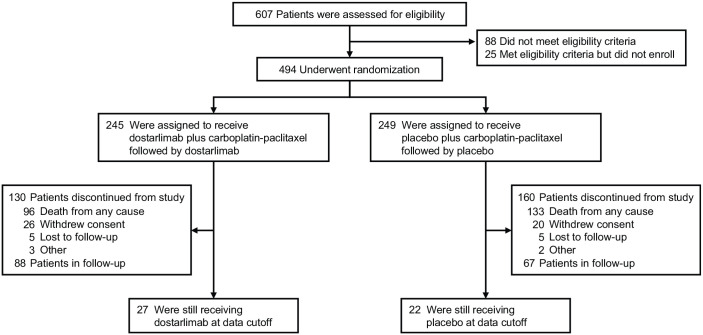
Enrollment and randomization of patients. CR, complete response; RECIST v1.1, Response Evaluation Criteria in Solid Tumors version 1.1.

At the start of cycle 7 (monotherapy period), 184 of 241 patients (76.3%) remained on treatment in the dostarlimab arm, and 184 of 246 patients (74.8%) remained on treatment in the placebo arm. As of the data cutoff date (September 22, 2023), 27 patients were receiving dostarlimab and 22 patients were receiving placebo; the most common reason for treatment discontinuation in both arms was disease progression (Figure 1).

Overall, the median duration of carboplatin and paclitaxel treatment in both arms was 18.0 (range: 17.9–19.0) weeks. In the chemotherapy period (cycles 1–6), median duration of dostarlimab or placebo treatment was 18.0 weeks in both arms (dostarlimab arm range: 3.0–29.0 weeks; placebo arm range: 2.1–28.0 weeks). In the monotherapy period (cycle 7 and beyond), median duration of dostarlimab treatment was 36.1 weeks (range: 6.0–174.9 weeks) and median duration of placebo treatment was 24.0 weeks (range: 6.0–175.1 weeks). Across the trial, overall median duration of dostarlimab treatment was 43.0 weeks (range: 3.0–192.6 weeks), whereas median duration of placebo treatment was 36.0 weeks (range: 2.1–193.1 weeks).

### Adverse events

A summary of TEAEs is provided in Table 1. TEAEs were experienced by 100% of patients in both arms; grade ⩾3 TEAEs were experienced by 72.2% of patients in the dostarlimab arm and 60.2% in the placebo arm. During the chemotherapy period (cycles 1–6), rates of TEAEs and grade ⩾3 TEAEs were similar between the dostarlimab and placebo arms (rates of TEAEs: 99.6% vs 100% and grade ⩾3 TEAEs 57.3% vs 51.2%, respectively). During the monotherapy period (cycle 7 and beyond), rates of TEAEs were similar between arms (89.7% vs 84.2%), whereas grade ⩾3 TEAEs were reported at a higher frequency in the dostarlimab arm than in the placebo arm (42.2% vs 23.9%). Across the duration of the trial, serious AEs occurred in 39.8% of patients in the dostarlimab arm and 28.0% in the placebo arm. The occurrence of serious TRAEs was approximately 7% higher with dostarlimab (19.5%) than with placebo (12.2%).

**Table 1.** Summary of safety outcomes in the RUBY trial (safety analysis set).

	Dostarlimab plus carboplatin–paclitaxel (*N* = 241)	Placebo plus carboplatin–paclitaxel (*N* = 246)
Any TEAE	241 (100)	246 (100)
Any TRAE	236 (97.9)	243 (98.8)
TEAE related to dostarlimab or placebo only	148 (61.4)	103 (41.9)
Grade ⩾3 TEAE	174 (72.2)	148 (60.2)
Grade ⩾3 TRAE	128 (53.1)	115 (46.7)
Serious TEAE	96 (39.8)	69 (28.0)
Serious TRAE	47 (19.5)	30 (12.2)
TEAE leading to discontinuation of dostarlimab or placebo	46 (19.1)	20 (8.1)
TEAE leading to discontinuation of carboplatin	20 (8.3)	15 (6.1)
TEAE leading to discontinuation of paclitaxel	26 (10.8)	25 (10.2)
TEAE leading to death	5 (2.1)^a^	0
TRAE leading to death	2 (0.8)^b^	0
Any irAE	141 (58.5)	91 (37.0)
irAE related to dostarlimab or placebo	98 (40.7)	40 (16.3)
Grade ⩾3 irAE	42 (17.4)	15 (6.1)
Grade ⩾3 irAE related to dostarlimab or placebo	32 (13.3)	8 (3.3)
Serious irAE	15 (6.2)	6 (2.4)
irAE leading to discontinuation of dostarlimab or placebo	21 (8.7)	8 (3.3)
irAE leading to death	0	0
Duration of overall treatment (weeks), median (range)	43.0 (3.0–192.6)	36.0 (2.1–193.1)

Data are shown as *n* (%) unless otherwise stated. TEAEs were defined as any event that was not present before the initiation of study treatment or any event already present that worsened in either intensity or frequency after exposure to study treatment. TRAEs were defined as an AE for which the investigator classified the relationship to study treatment as “Yes.” irAEs were defined as events from a predefined list occurring at grade 2 and above.

a Three deaths were not related to study treatment (opiate overdose, COVID-19, and general physical health deterioration).

b One death was considered by the investigator as related to dostarlimab, carboplatin, and paclitaxel and occurred during the first 6 cycles (myelosuppression); one death was considered related to dostarlimab and occurred during the 90-day safety follow-up (hypovolemic shock).

AE, adverse event; COVID-19, coronavirus disease 2019; irAE, immune-related adverse event; TEAE, treatment-emergent adverse event; TRAE, treatment-related adverse event.

Overall, 24.9% of patients in the dostarlimab arm and 16.3% of patients in the placebo arm discontinued study treatment due to an AE. Discontinuation rates of any study treatment were more common for both the dostarlimab and placebo arms during the chemotherapy period (16.6% and 13.8%, respectively) compared with the monotherapy period (11.4% and 3.3%, respectively). The occurrence of TEAEs resulting in carboplatin discontinuation (dostarlimab arm, 8.3%; placebo arm, 6.1%) or paclitaxel discontinuation (dostarlimab arm, 10.8%; placebo arm, 10.2%) was similar between arms. TEAEs led to discontinuation of dostarlimab or placebo in 19.1% and 8.1% of patients, respectively. The most common TEAEs leading to discontinuation of dostarlimab or placebo are reported in Table 2.

**Table 2.** The most common TEAEs leading to discontinuation of dostarlimab or placebo.

	Dostarlimab plus Carboplatin–paclitaxel (*N* = 241)	Placebo plus Carboplatin–paclitaxel (*N* = 246)
Discontinuation of any study treatment over all cycles	60 (24.9)	40 (16.3)
TEAEs leading to discontinuation in ⩾2% of either arm over all cycles		
Infusion-related reaction	5 (2.1)	8 (3.3)
Peripheral neuropathy	5 (2.1)	7 (2.8)
Peripheral sensory neuropathy	7 (2.9)	1 (0.4)
Discontinuation of dostarlimab or placebo over all cycles	46 (19.1)	20 (8.1)
TEAEs leading to discontinuation of dostarlimab or placebo in ⩾1% of either arm over all cycles		
Infusion-related reaction	3 (1.2)	1 (0.4)
Thrombocytopenia	1 (0.4)	3 (1.2)
Maculopapular rash	3 (1.2)	0
Discontinuation of any study treatment by treatment period		
Cycles 1–6 (chemotherapy period)	40 (16.6)	34 (13.8)
Cycles ⩾7 (monotherapy period)	21 (11.4)	6 (3.3)

Data are shown as *n* (%).

TEAE, treatment-emergent adverse event.

### Treatment-related adverse events

**Figure 2.**
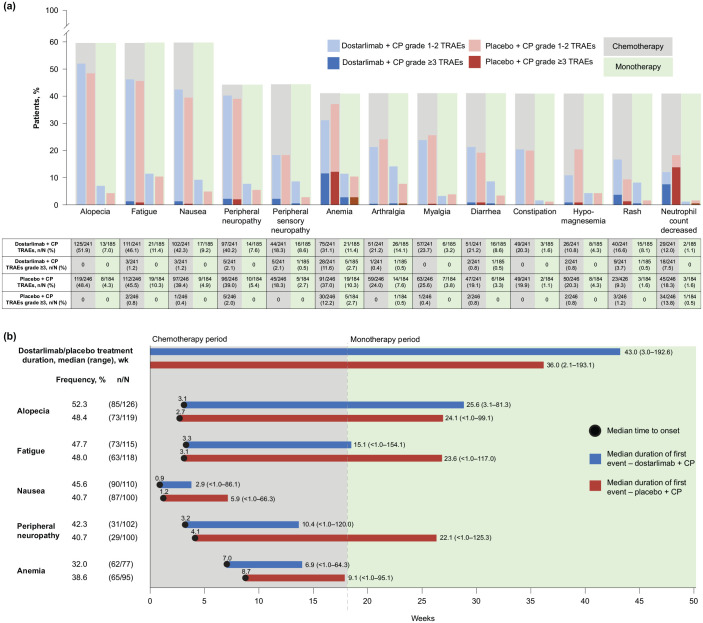
Occurrence, onset, and duration of TRAEs. (a) Common TRAEs (⩾15% in either arm) by treatment period and grade. (b) Onset and duration of the most frequent TRAEs (occurring in ⩾30% of patients in either treatment arm) (safety analysis set). In panel B, *n*/*N* represents the number of patients with duration data over the number of patients with onset data. Patients with duration data had at least 1 day between the resolution of all events from the first course of the TRAE to the onset of a second course of the AE. Patients without duration data did not fit this criterion. The duration was defined as time from onset of any TRAE considered in this analysis to the first time the patient was free of any such AE. Median onset was the median time to onset of the first occurrence of the specified AE. AE, adverse event; CP, carboplatin–paclitaxel; irAE, immune-related AE; TRAE, treatment-related adverse event.

TRAEs that occurred in at least 15% of patients in either arm occurred at similar rates between arms and were mostly low grade (Figure 2(a)). These TRAEs occurred more often in the chemotherapy period than in the monotherapy period. Treatment-related hypomagnesemia was experienced by a higher percentage of patients in the placebo arm than in the dostarlimab arm during the chemotherapy period (20.3% in the placebo arm vs 10.8% in the dostarlimab arm), although 4.3% of patients in both arms experienced hypomagnesemia in the monotherapy period. Treatment-related rash was experienced by a higher percentage of patients in the dostarlimab arm than in the placebo arm during both the chemotherapy period (16.6% in the dostarlimab arm vs 9.3% in the placebo arm) and the monotherapy period (8.1% in the dostarlimab arm vs 1.6% in the placebo arm). No increases in the severity of TRAEs were noted in the dostarlimab arm when compared with the placebo arm. Few TRAE events occurred after 24 months of treatment; to note, at 25 months, the safety population consisted of 53 patients in the dostarlimab arm and 38 patients in the placebo arm.

For TRAEs seen in more than 30% of patients in either arm, the median onset of those TRAEs in patients with AE duration data (patients having at least 1 day between the resolution of all events from the first course of the AE to the onset of a second course of the AE) fell within the chemotherapy period (Figure 2(b)). No meaningful differences in the onset or duration of these individual TRAEs were observed. Median onset for nausea for both arms was within the first 3 weeks, which corresponds to the first cycle of treatment, whereas the median onset for fatigue and peripheral neuropathy was within the second 3 weeks (cycle 2). Median onset of anemia was slightly later than the other TRAEs examined, with a median onset observed at cycle 3 (7.0 weeks in the dostarlimab arm and 8.7 weeks in the placebo arm).

Five deaths due to AEs were reported, all were in the dostarlimab arm. One death (myelosuppression) was reported by the investigator as related to dostarlimab, carboplatin, and paclitaxel and occurred during the first six cycles, in the chemotherapy phase. One death (hypovolemic shock) was related to dostarlimab and occurred during the 90-day safety follow-up after the last dose. Three deaths were deemed not related to study treatment: opiate overdose, COVID-19, and general deterioration of physical health. No deaths due to AEs were reported in the placebo arm.

### Immune-related adverse events

TE irAEs occurred in 58.5% of patients in the dostarlimab arm and in 37.0% of patients in the placebo arm (Table 1). The most common TE irAEs (occurring in ⩾5% of patients in either treatment arm) were arthralgia, infusion-related reactions, hypothyroidism, rash, maculopapular rash, alanine aminotransferase (ALT) increased, pruritus, and aspartate aminotransferase (AST) increased (Table 3). Although most of these TE irAEs were more frequent in the dostarlimab arm than the placebo arm, arthralgia and infusion-related reactions occurred at a similar frequency in both arms (arthralgia, 14.9% vs 13.0%; infusion-related reactions, 12.9% vs 12.2%). Few patients discontinued dostarlimab or placebo because of TE irAEs (8.7% in the dostarlimab arm and 3.3% in the placebo arm), and there were no TE irAE-related deaths.

**Table 3.** Frequency and severity of irAEs occurring in ⩾5% of patients in either treatment arm (safety analysis set).

	Dostarlimab plus carboplatin–paclitaxel (*N* = 241)	Placebo plus carboplatin–paclitaxel (*N* = 246)
Arthralgia		
Any grade/grade ⩾3 TE irAE	36 (14.9)/3 (1.2)	32 (13.0)/1 (0.4)
Any grade/grade ⩾3 irAE related to dostarlimab or placebo	16 (6.6)/1 (0.4)	16 (6.5)/1 (0.4)
Serious irAE	0	0
irAE leading to discontinuation of dostarlimab or placebo	2 (0.8)	0
Infusion-related reaction		
Any grade/grade ⩾3 TE irAE	31 (12.9)/2 (0.8)	30 (12.2)/3 (1.2)
Any grade/grade ⩾3 irAE related to dostarlimab or placebo	4 (1.7)/0	0/0
Serious irAE	2 (0.8)	0
irAE leading to discontinuation of dostarlimab or placebo	3 (1.2)	1 (0.4)
Hypothyroidism		
Any grade/grade ⩾3 TE irAE	29 (12.0)/0	8 (3.3)/1 (0.4)
Any grade/grade ⩾3 irAE related to dostarlimab or placebo	29 (12.0)/0	7 (2.8)/1 (0.4)
Serious irAE	0	1 (0.4)
irAE leading to discontinuation of dostarlimab or placebo	1 (0.4)	0
Rash		
Any grade/grade ⩾3 TE irAE	22 (9.1)/11 (4.6)	6 (2.4)/3 (1.2)
Any grade/grade ⩾3 irAE related to dostarlimab or placebo	17 (7.1)/10 (4.1)	5 (2.0)/3 (1.2)
Serious irAE	2 (0.8)	0
irAE leading to discontinuation of dostarlimab or placebo	0	1 (0.4)
Maculopapular rash		
Any grade/grade ⩾3 TE irAE	17 (7.1)/6 (2.5)	0/0
Any grade/grade ⩾3 irAE related to dostarlimab or placebo	12 (5.0)/6 (2.5)	0/0
Serious irAE	0	0
irAE leading to discontinuation of dostarlimab or placebo	3 (1.2)	0
ALT increased		
Any grade/grade ⩾3 TE irAE	15 (6.2)/5 (2.1)	4 (1.6)/1 (0.4)
Any grade/grade ⩾3 irAE related to dostarlimab or placebo	15 (6.2)/5 (2.1)	3 (1.2)/0
Serious irAE	0	0
irAE leading to discontinuation of dostarlimab or placebo	2 (0.8)	1 (0.4)
Pruritus		
Any grade/grade ⩾3 TE irAE	16 (6.6)/1 (0.4)	4 (1.6)/0
Any grade/grade ⩾3 irAE related to dostarlimab or placebo	8 (3.3)/1 (0.4)	3 (1.2)/0
Serious irAE	0	0
irAE leading to discontinuation of dostarlimab or placebo	1 (0.4)	0
AST increased		
Any grade/grade ⩾3 TE irAE	12 (5.0)/5 (2.1)	3 (1.2)/2 (0.8)
Any grade/grade ⩾3 irAE related to dostarlimab or placebo	10 (4.1)/5 (2.1)	2 (0.8)/1 (0.4)
Serious irAE	0	0
irAE leading to discontinuation of dostarlimab or placebo	2 (0.8)	0

Data are shown as *n* (%). irAEs were defined as events from a predefined list occurring at grade 2 and above.

ALT, alanine aminotransferase; AST, aspartate aminotransferase; irAE, immune-related adverse event; TE, treatment-emergent.

Dostarlimab- or placebo-related irAEs were reported in 40.7% of patients in the dostarlimab arm and 16.3% of patients in the placebo arm. Grade ⩾3 dostarlimab- or placebo-related irAEs were reported for 13.3% of patients in the dostarlimab arm and for 3.3% of patients in the placebo arm. The most common dostarlimab- or placebo-related irAEs (⩾5% of patients in either arm) were hypothyroidism, rash, arthralgia, and ALT increased (Table 3). Dostarlimab- or placebo-related hypothyroidism occurred in 12.0% of patients in the dostarlimab arm and in 2.8% of patients in the placebo arm. In the dostarlimab arm, no hypothyroidism events were grade ⩾3, and only one event led to discontinuation. Dostarlimab- or placebo-related rash and ALT increased also occurred more frequently in the dostarlimab arm than in the placebo arm (rash, 7.1% vs 2.0%; ALT increased, 6.2% vs 1.2%). Approximately half of the reported arthralgia events were considered to be dostarlimab- or placebo-related, with a similar frequency in the dostarlimab (6.6%) and placebo (6.5%) arms.

**Figure 3.**
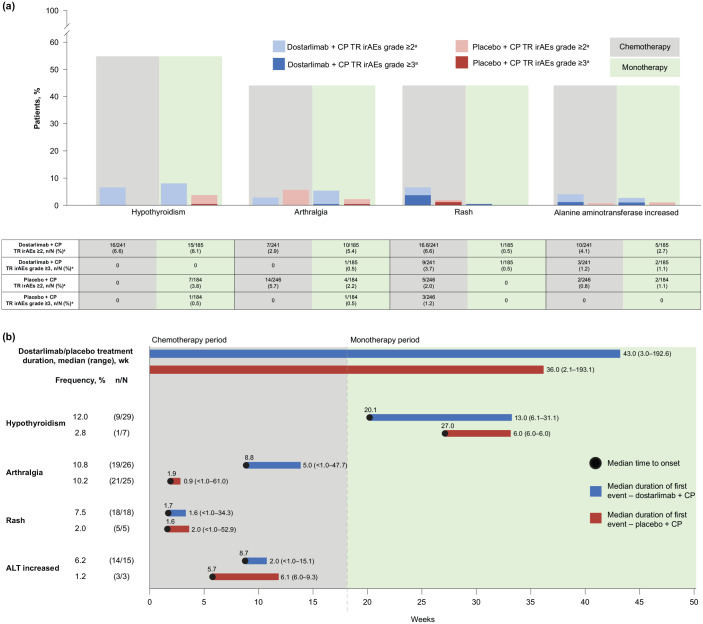
Occurrence, onset, and duration of dostarlimab- or placebo-related irAEs. (a) Most common dostarlimab- or placebo-related irAEs (occurring in ⩾5% of patients in either treatment arm) by treatment period and grade. (b) Onset and duration of the most frequent dostarlimab- or placebo-related irAEs (occurring in ⩾5% of patients in either treatment arm). IrAEs were defined as events from a predefined list occurring at grade 2 and above. In panel B, *n*/*N* represents the number of patients with duration data over the number of patients with onset data. Patients with duration data had at least 1 day between the resolution of all events from the first course of the irAE to the onset of a second course of the irAE. Patients without duration data did not fit this criterion. The duration was defined as time from onset of any irAE considered in this analysis to the first time the patient was free of any such irAE; a gap of at least 1 day between the resolution of all irAEs from the first course to the onset of second course was required. Median onset was the median time to onset of the first occurrence of the specified irAE. ^a^TR irAE refers to dostarlimab- or placebo-related irAEs. AE, adverse event; ALT, alanine aminotransferase; CP, carboplatin–paclitaxel; irAE, immune-related adverse event; TR, treatment-related.

Occurrence of dostarlimab- or placebo-related irAEs by treatment period and grade is shown in Figure 3(a). In the dostarlimab arm, 68 of 241 patients (28.2%) experienced a dostarlimab- or placebo-related irAE during the chemotherapy period (17 of 241 (7.1%) experienced grade ⩾3 events), and 48 of 185 patients (25.9%) experienced a TR irAE during the monotherapy period (16 of 185 (8.6%) experienced grade ⩾3 events). In the placebo arm, 28 of 246 patients (11.4%) experienced a dostarlimab- or placebo-related irAE during the chemotherapy period (4 of 246 (1.6%) experienced grade ⩾3 events), and 16 of 184 patients (8.7%) experienced a dostarlimab- or placebo-related irAE during the monotherapy period (4 of 184 (2.2%) experienced grade ⩾3 events). Among the most frequent (⩾5% of patients in either arm) dostarlimab- or placebo-related irAEs, most events of hypothyroidism, rash, arthralgia, and ALT increased were grade 2 in severity (Figure 3(a)). With the exception of hypothyroidism, the median onset of the most frequent dostarlimab- or placebo-related irAEs in patients with duration data (patients having at least 1 day between the resolution of all events from the first course of the AE to the onset of a second course of the AE) had a median onset during the chemotherapy treatment period (Figure 3(b)). Treatment-related arthralgia and ALT increased occurred throughout the trial in both treatment arms. Treatment-related hypothyroidism and treatment-related rash presented in both treatment periods in the dostarlimab arm. In the placebo arm, treatment-related hypothyroidism presented only in the monotherapy period whereas treatment-related rash presented only in the chemotherapy period.

### Management of irAEs

TE irAE management is summarized in Table 4. Infusion delays and discontinuations were infrequent in patients with irAEs in either arm. In the dostarlimab arm, irAEs led to dostarlimab infusion delays in 11.6% of patients and to dostarlimab discontinuation in 8.7% of patients. In the placebo arm, irAEs led to placebo infusion delays in 3.3% of patients and to placebo discontinuation in 3.3% of patients.

**Table 4.** Summary of TE irAE management (safety analysis set).

	Dostarlimab plus carboplatin–paclitaxel (*N* = 241)	Placebo plus carboplatin–paclitaxel (*N* = 246)
Patients with any irAE	141 (58.5)	91 (37.0)
Patients with an irAE leading to dostarlimab/placebo infusion delay	28 (11.6)	8 (3.3)
Patients with an irAE leading to dostarlimab/placebo discontinuation	21 (8.7)	8 (3.3)
Patients treated with steroid^a^	77 (54.6)	48 (52.7)
Patients treated with high-dose steroids^a,b^	38 (27.0)	22 (24.2)
Patients treated with thyroid therapy/anti-thyroid therapy^a^	28 (19.9)	6 (6.6)
Patients treated with IMM^a^	91 (64.5)	52 (57.1)
Resolution of events after treatment^c^	66 (72.5)	43 (82.7)
Patients not treated with IMM^a^	50 (35.5)	39 (42.9)
Resolution of events^d^	39 (78.0)	28 (71.8)

Data are shown as *n* (%). IrAEs were defined as events from a predefined list occurring at grade 2 and above.

a Percentages calculated based on the number of patients with irAEs.

b High-dose steroids were defined as 40 mg/day of prednisone/prednisolone and/or 200 mg/day of cortisone and/or 160 mg/day of hydrocortisone and/or 32 mg/day of triamcinolone/methylprednisolone.

c Percentages calculated based on the number of patients who were treated with IMM.

d Percentages calculated based on the number of patients who were not treated with IMM.

IMM, immunomodulatory medication; irAE, immune-related adverse event; TE, treatment-emergent.

In patients experiencing irAEs in the dostarlimab arm, 54.6% of patients were treated with steroids, and 19.9% of patients were treated with thyroid or anti-thyroid therapy. Overall, 64.5% of patients in the dostarlimab arm who experienced an irAE were treated with an immunomodulatory agent (defined as systemic corticosteroids, immunosuppressants, immunostimulants, thyroid, and anti-thyroid treatments); 72.5% of these patients experienced resolution after treatment. Of those patients experiencing an irAE who were not treated with an immunomodulatory agent (35.5%), 78.0% experienced resolution. In patients experiencing irAEs in the placebo arm, 52.7% of patients were treated with steroids, and 6.6% of patients were treated with thyroid or anti-thyroid therapy. Overall, 57.1% of patients in the placebo arm who experienced an irAE were treated with an immunomodulatory agent; 82.7% of these patients experienced resolution after treatment. Of those patients experiencing an irAE in the placebo arm who were not treated with an immunomodulatory agent (42.9%), 71.8% experienced resolution.

## Discussion

Overall, the results of the RUBY trial demonstrate that dostarlimab plus CP has a favorable benefit–risk profile that makes it a valuable treatment option for patients with primary advanced or recurrent EC. The safety profile of dostarlimab plus CP was generally consistent with that of the individual components.

Concurrent administration of dostarlimab with chemotherapy did not affect the number of patients who discontinued any treatment during the chemotherapy period (cycles 1–6); concordant with this observation, a similar percentage of patients in the dostarlimab arm (76.3%) remained in the study at the start of cycle 7 compared with the placebo arm (74.8%). Similar rates of discontinuation of carboplatin or paclitaxel were seen in both the dostarlimab plus CP and placebo plus CP arms.

Furthermore, given the similar median duration of chemotherapy between the two arms, the addition of dostarlimab did not compromise the receipt of chemotherapy. The most common TRAEs and TR irAEs occurred more frequently during the chemotherapy period of treatment.

The AE profile observed in the dostarlimab arm was generally observed to be similar to that of the anti-PD-(L)1 class of therapy in combination with chemotherapy. However, it is challenging to perform inter-trial comparisons. One limitation to comparing AEs between trials is the reporting requirements. The RUBY trial, per protocol specifications, reported all AEs, whereas other trials may not report specific AEs that fall below an indicated reporting threshold.^25,26^ By reporting all AEs, the RUBY trial results present a comprehensive look at the safety of the combination treatment. In addition, beyond the difference in reporting of TEAEs, trials will have an individually defined set of AEs that were identified as irAEs, which unfortunately prevents direct irAE comparison between trials.

Chemotherapy did not appear to exacerbate the irAEs associated with dostarlimab, and few patients discontinued dostarlimab because of an irAE (7.9%). Although in Part 1 of the RUBY trial dostarlimab is given in combination with chemotherapy, the most common dostarlimab-related irAEs were seen at similar rates to those observed in the GARNET study of dostarlimab monotherapy: hypothyroidism (12.0% in the RUBY trial and 8.3% in the GARNET trial), arthralgia (6.6% and 3.2%, respectively), and increased ALT (6.2% and 2.5%, respectively).^22^ Rash was observed more frequently in the RUBY trial than in the GARNET trial (7.1% and 1.6%, respectively); however, rash is known to be associated with both immunotherapy and chemotherapy, and was observed more frequently during the chemotherapy period of treatment. Notably, in the RUBY trial, all patients experienced resolution (median duration of rash was 1.6 weeks); 10 patients (4.1%) experienced grade 3 events. The durations of irAEs and the rates of resolution of irAEs were generally similar between the dostarlimab arm and the placebo arm, with most irAEs resolving. In addition, in both trial arms, the guidelines for management resulted in high rates of resolution for most irAEs, providing evidence that these guidelines are of use in irAE management with immune-checkpoint inhibitor therapies in clinical practice.^21^

This analysis of safety from Part 1 of the RUBY trial details the timing and resolution of AEs and irAEs, an area for which little information is known for immunotherapy and chemotherapy combinations in EC. In addition, this analysis applies an objective approach to the definition of irAEs, which potentially minimizes bias from investigators in the analysis of irAEs. However, although the investigators did not influence how irAEs were reported, investigators did decide how irAEs were treated, and some irAEs reportedly related to placebo may not have needed treatment for management. Therefore, there was a risk of bias that may have led to the overtreatment of some of the observed irAEs. To note, the management of some AEs requires a similar approach regardless of whether they were related to immunotherapy or chemotherapy.^27,28^ Importantly, the reported safety results from Part 1 of the RUBY trial help to provide better insight to clinicians on when AEs occur, which in turn helps clinicians to advise their patients better. The need for long-term safety monitoring, which is essential to optimizing outcomes with immunotherapy, provides valuable information given that immunotherapy and chemotherapy combinations are relatively new treatment options in EC.

In conclusion, these safety data further support that dostarlimab plus CP has a favorable benefit–risk profile and provide valuable information to support clinical understanding of this new standard of care for patients with primary advanced or recurrent EC.

## Supplemental Material

sj-pdf-1-tam-10.1177_17588359241277656 – Supplemental material for Safety of dostarlimab in combination with chemotherapy in patients with primary advanced or recurrent endometrial cancer in a phase III, randomized, placebo-controlled trial (ENGOT-EN6-NSGO/GOG-3031/RUBY) Supplemental material, sj-pdf-1-tam-10.1177_17588359241277656 for Safety of dostarlimab in combination with chemotherapy in patients with primary advanced or recurrent endometrial cancer in a phase III, randomized, placebo-controlled trial (ENGOT-EN6-NSGO/GOG-3031/RUBY) by Annika Auranen, Matthew A. Powell, Vladyslav Sukhin, Lisa M. Landrum, Graziana Ronzino, Joseph Buscema, Dirk Bauerschlag, Roy Lalisang, David Bender, Lucy Gilbert, Amy Armstrong, Tamar Safra, Nicole Nevadunsky, Alexandra Sebastianelli, Brian Slomovitz, Kari Ring, Robert Coleman, Iwona Podzielinski, Ashley Stuckey, Michael Teneriello, Sarah Gill, Bhavana Pothuri, Lyndsay Willmott, Sudarshan Sharma, Christine Dabrowski, Grace Antony, Shadi Stevens, Mansoor Raza Mirza and Evelyn Fleming in Therapeutic Advances in Medical Oncology

sj-pdf-2-tam-10.1177_17588359241277656 – Supplemental material for Safety of dostarlimab in combination with chemotherapy in patients with primary advanced or recurrent endometrial cancer in a phase III, randomized, placebo-controlled trial (ENGOT-EN6-NSGO/GOG-3031/RUBY) Supplemental material, sj-pdf-2-tam-10.1177_17588359241277656 for Safety of dostarlimab in combination with chemotherapy in patients with primary advanced or recurrent endometrial cancer in a phase III, randomized, placebo-controlled trial (ENGOT-EN6-NSGO/GOG-3031/RUBY) by Annika Auranen, Matthew A. Powell, Vladyslav Sukhin, Lisa M. Landrum, Graziana Ronzino, Joseph Buscema, Dirk Bauerschlag, Roy Lalisang, David Bender, Lucy Gilbert, Amy Armstrong, Tamar Safra, Nicole Nevadunsky, Alexandra Sebastianelli, Brian Slomovitz, Kari Ring, Robert Coleman, Iwona Podzielinski, Ashley Stuckey, Michael Teneriello, Sarah Gill, Bhavana Pothuri, Lyndsay Willmott, Sudarshan Sharma, Christine Dabrowski, Grace Antony, Shadi Stevens, Mansoor Raza Mirza and Evelyn Fleming in Therapeutic Advances in Medical Oncology

## Declarations

**Ethics approval and consent to participate:** The trial is approved by the institutional review board at each study site and conducted in accordance with the Declaration of Helsinki and Good Clinical Practice Guidelines of the International Council for Harmonisation. All patients provided written informed consent for participation.

**Consent for publication:** Not applicable.

**Availability of data and materials:** GSK is committed to sharing anonymized subject-level data from interventional trials as per GSK policies and as applicable. Requests for subject-level data should be done via the GSK link: https://www.gsk-studyregister.com/en/.

**Prior presentation:** Portions of the data reported in this manuscript were previously reported at the 2023 American Society of Clinical Oncology Annual Meeting (June 2–6; Chicago, IL, USA) and the 2023 European Society for Medical Oncology Congress (October 20–24; Madrid, Spain).
