# Supplementary material for: Safety of dostarlimab in combination with chemotherapy in patients with primary advanced or recurrent endometrial cancer in a phase III, randomized, placebo-controlled trial (ENGOT-EN6-NSGO/GOG-3031/RUBY)
Source: Ther Adv Med Oncol. 2024 Sep 28;16:17588359241277656. doi: 10.1177/17588359241277656 (PMC11439170; doi:10.1177/17588359241277656)
Supplement: sj-pdf-1-tam-10.1177_17588359241277656 – Supplemental material for Safety of dostarlimab in combination with chemotherapy in patients with primary advanced or recurrent endometrial cancer in a phase III, randomized, placebo-controlled trial (ENGOT-EN6-NSGO/GOG-3031/RUBY) [file sj-pdf-1-tam-10.1177_17588359241277656.pdf]

PROTOCOL APPROVAL WITH MODIFICATIONS

**DATE:**	13 Jun 2019
**TO:**	Amanda Diven
**PROTOCOL:**	TESARO, Inc - 4010-03-001 / ENGOT EN-6 / GOG-3031, A phase 3, randomized, double-blind, multicenter study of dostarlimab (TSR-042) plus carboplatin-paclitaxel versus placebo plus carboplatin-paclitaxel in patients with recurrent or primary advanced endometrial cancer (RUBY) (Pro00033913)
**APPROVAL DATE:**	5 Jun 2019
**EXPIRATION DATE:**	5 Jun 2020

IRB APPROVED DOCUMENTATION:

Protocol Version:

- Protocol (Version 1.1, Dated 13 March 2019)

Consent Template:

- Informed Consent Form And Authorization To Disclose Health Information (Advarra IRB Approved Version 13 Jun 2019)

Product Information:

- Package Insert for Carboplatin Injection (Dated 03/14/2012)
- Package Insert for Paclitaxel Injection, USP (Rev. June 2013)
- Investigator's Brochure for Dostarlimab (Also known as TSR-042) (Edition No.: 4.0, Dated 12 March 2019)

Other Material:

- Clinical Study Participant Emergency Card 4010-03-001 Master v1.0 16Apr2019

The IRB approved the above referenced protocol with the modifications listed below on 5 Jun 2019:

- **Modifications to the Informed Consent Form**

If you wish to have the IRB reconsider the imposed modifications, you may follow the procedures outlined below:

1. Submit supporting documentation that addresses the IRB's concerns.
2. Provide a written justification for relief of any IRB imposed condition.

On 13 Jun 2019, the IRB reviewed and approved the additional revisions to the Informed Consent Form.

Please Note: Each Principal Investigator will receive a separate IRB Approval notice allowing them to conduct the study.

Subpart D Determination – Newborn follow-up
21 CFR 50.51 / 45 CFR 46.404
This protocol plans to collect data regarding the newborn child born to a female participant on the clinical trial. The Board performed a Subpart D analysis and determined that this follow-up portion of the study presents no more than minimal risk (21 CFR 50.51/45 CFR 46.404). The Board requires that one parent or the legal guardian provide permission for the minor to participate in the research. Assent from the minor participant is not required due to infancy.

Subpart B Determination – Pregnancy follow-up
45 CFR §46.204
This protocol plans to collect data on the pregnancy of female participants on the clinical trial and the Board determined that the research meets requirements under Subpart B. The Board determined that this follow-up portion of the research presents no more than minimal risk, there is no prospect of direct benefit to the woman or the fetus, and the purpose of the research is the development of important biomedical knowledge which cannot be obtained by any other means. The Board requires that consent to participate in this research study be obtained only from the pregnant woman.

The above referenced material is available on your Advarra CIRBI Platform under the "IRB Issued Documents" tab.

If there are any changes to the IRB approved material, IRB approval will be needed prior to use. This includes changes in relative size and type of font in the material to be viewed by potential subjects.

If the study is expected to last beyond the approval period, you must request and receive re-approval prior to the expiration date noted above. A report to the Board on the status of this study is due prior to the expiration date or at the time the study closes, whichever is earlier. It is recommended that you submit status reports at least 4 weeks prior to your expiration date to avoid any additional fees or lapses in approval.

Approved investigators and sites are required to submit to Advarra for review, and await a response prior to implementing, any amendments or changes in the protocol; informed consents; advertisements or recruitment materials ("study-related materials"); investigators; or sites (primary and additional).

Approved investigators and sites are required to notify Advarra of the following reportable events, including, but not limited to: unanticipated problems involving risks to subjects or others; unanticipated adverse device effects; protocol violations that may affect the subjects' rights, safety, or well-being and/or the completeness, accuracy and reliability of the study data; subject death; suspension of enrollment; or termination of the study.

Please review the IRB Handbook located in the "Reference Materials" section of Advarra CIRBI™ Platform (www.cirbi.net). A copy of the most recent IRB roster is also available.

Thank you for selecting Advarra IRB to provide oversight for your research project.

> 6940 Columbia Gateway Dr., Suite 110 410-884-2900 OFFICE ADVARRA.COM
Columbia, MD 21046
